# Efficacy of omental graft to facilitate canine radial fracture repair

**DOI:** 10.18849/ve.v8i4.665

**Published:** 2023-11-08

**Authors:** Rhyanna Dietrich+

**Keywords:** CANINE, DOGS, FRACTURE, HEALING TIME, OMENTUM, RADIUS

## Abstract

**PICO question:**

In dogs with a displaced radial fracture, does the use of a free autologous greater omental graft, combined with other standard fracture repair methods, compared to not using a greater omental graft, reduce fracture healing time?

**Clinical bottom line:**

**Category of research:**

Treatment.

**Number and type of study designs reviewed:**

Three papers were critically reviewed: one retrospective clinical study and two experimental case control studies. All three papers answered the PICO question. Each of the three papers had a very small sample size, with two having a sample size of 16 (n = 4 in the relevant experimental group, and n = 4 for the control). The third had an initial sample of 25 that was later reduced to 19, as six dogs were excluded from the study.

**Strength of evidence:**

Weak.

**Outcomes reported:**

Two papers were experimental case control studies, which looked at radial fracture healing of dogs that had undergone an osteotomy, followed by bone plate and screw fixation, as well as either with a free autologous greater omental graft (OG) or without. Healing was measured in both studies via radiographical analysis using a modified Lane and Sandhu scoring system, and histopathological analysis post euthanasia with Heiple’s histopathological scoring system. Both studies found higher radiographic and higher histopathological scores in the OG group, though there was a large overlap between group scores. There was no mention of randomisation or power analysis in either of these studies, and blinding was only mentioned regarding histopathological analysis.

The other was a retrospective study, looking at the outcomes of radial and ulna fractures in small breed dogs, after being surgically treated with a plate and screw, and either with or without OG. They found that dogs with omental grafts healed faster than those without, and had no major complications (whereas the non-OG group did). Note that this study was not (and could not) be randomised due to its nature, and it made no mention of blinding.

**Conclusion:**

All three studies concluded that the use of OG assisted healing in canine radial fractures. However, care must be taken when applying these results to practice, as the studies lack robustness. While two of the studies had a good study design, both had very small sample sizes and neither mentioned randomisation. Blinding was only mentioned in the histopathological analysis, not radiographical analysis, and while both studies reported significant differences between their respective OG and control groups, they failed to account for multiple comparisons in statistical analysis, which likely skewed the results. The third study also represents a weak level of evidence, due to its retrospective nature and other limitations. Its small sample size, and the fact that 4/8 control (non-OG) dogs received a different type of graft, contributed to this.

Due to the small number of animals in each study and the poor-quality design, it is concluded that there is weak evidence to support the PICO question. Further randomised blinded clinical trials with larger samples sizes are recommended, to increase the strength of the evidence before the routine clinical use of greater omentum grafts for aiding fracture repair can be recommended, considering that this means an additional abdominal surgical procedure.

## How to apply this evidence in practice

The application of evidence into practice should take into account multiple factors, not limited to: individual clinical expertise, patient’s circumstances and owners’ values, country, location or clinic where you work, the individual case in front of you, the availability of therapies and resources.

Knowledge Summaries are a resource to help reinforce or inform decision making. They do not override the responsibility or judgement of the practitioner to do what is best for the animal in their care.

## Clinical scenario

A working Border Collie arrives at the practice after being hit by a car. Radiographs show a displaced comminuted fracture in the left radius, midshaft. You recommend using a bone plate and screws to reduce and stabilise the fracture. You explain that healing may be delayed due to the complex nature of the fracture. The owner is worried the bone will not heal properly and that they will have to retire the dog. They rely on it to manage their sheep, so are hoping for a fast and complete recovery so the dog can return to work. They ask if there is anything further that can be done to speed up the healing. The veterinarian considers what they know about the use of omental grafts to improve bone healing, and whether an omental graft (OG) should be used in this situation to reduce the fracture healing time.

## The evidence

Three papers answered the PICO question, with two studies being experimental case control studies, (Bigham-Sadegh et al., 2012; and Karimi et al., 2013) and one being a retrospective clinical trial (Baltzer et al., 2015). All three studies had a small sample size, and none were randomised. The two experimental studies mentioned blinding for the histopathological analysis, however other than this, no study reported blinding in their methods. The two experimental case control studies did however have both an experimental group and a control group, which were kept as similar as possible, aside from the intervention being studied. They both compared fracture healing in medium sized dogs with (n = 4) or without (n = 4) assistance of OG, measured by radiographical and histopathological analysis. The retrospective study was understandably not able to do this due to the nature of the study, however significant differences within the control group (some with a cancellous or corticocancellous bone graft as an additional treatment) result in lack of confidence in the conclusions relating to the PICO question.

## Summary of the evidence

**Bigham-Sadegh et al. (2012):** 

Population:	Male adult medium sized (~25 kg) dogs (3–4 years old), free of infectious or parasitic illnesses.
Sample size:	16 dogs.
Intervention details:	All dogs (including controls) underwent aseptic surgery to remove a 30 x 30 mm piece of omentum from the free end of the greater omentum. All dogs also underwent a midshaft radial osteotomy, where a 10 mm transverse bone defect was created with an electrical bone-cutting saw. This was followed by fracture fixation with a plate and screws and one of the following interventions: free greater omental graft (OG) (experimental, n = 4 dogs); OG and 1 ml autologous freshly cultured adipose-derived adult stem cells in regular media (experimental, n = 4 dogs); and OG and 1 ml regular culture medium (experimental, n = 4 dogs); or no additional treatment (control, n = 4 dogs). All dogs were euthanised and had bone harvested at week 8.
Study design:	Experimental case control study.
Outcome studied:	Fracture repair: Radiographic evaluation of bone formation, union and remodelling using modified Lane and Sandhu scoring system (Lane & Sandhu, 1987), on day 1, and week 2, 4, 6 and 8. Histological evaluation of bone union, cancellous bone, cortical bone and marrow using Heiple’s histopathological scoring system (Heiple et al., 1987) after euthanasia at week 8.
Main Findings (relevant to PICO question)	Higher radiographic scores in the OG only group compared to the control group at week 6 and 8. Mean radiographic scores were similar (week 6 OG 5/10 vs control 3/10, week 8 OG 6/10 vs control 4/10), and there was a large amount of overlap between groups. Higher histopathology scores for union and cortical bone in OG only group compared to control group at week 8. For cancellous bone and marrow, the OG only group had higher histopathology scores, however there was significant overlap between groups.
Limitations:	Very small sample size. There is no sample heterogeneity. Unable to examine histologically throughout the study due to ethics constraints. A healthy dog population does not reflect hospitalised dogs. An osteotomy may not reflect natural fractures. Multiple comparisons in statistical analysis not accounted for. No power analysis. All authors worked in the university that conducted the study and received financial support by the university. There is potential for bias. This study does not mention who conducted the statistical analysis or who estimated the radiographs. If the authors were involved, there is potential for bias.

**Karimi et al. (2013):** 

Population:	Adult medium sized (~26kg) male dogs, free of evident infectious or parasitic illness.
Sample size:	16 dogs.
Intervention details:	All dogs (including controls) underwent aseptic surgery to remove a 30 x 30 mm piece of omentum from the free end of the greater omentum. All dogs also underwent a midshaft radial osteotomy, where a 10 mm transverse bone defect was created using an electrical bone-cutting saw. This was followed by fracture fixation with a plate and screws and one of the following interventions: a greater omentum graft (OG) (experimental, n = 4 dogs); the defect filled with a segment of coral and covered by OG (experimental, n = 4 dogs); the defect filled with a segment of coral (experimental, n = 4 dogs); or no additional treatment (control, n = 4 dogs). All dogs were euthanised and had bone harvested at day 60.
Study design:	Experimental case control study.
Outcome studied:	Radiographic evaluation of bone formation, union and remodelling using modified Lane and Sandhu scoring system (Lane & Sandhu, 1987), on day 1, 30 and 60. Histological evaluation of bone union, cancellous bone, cortical bone and marrow using Heiple’s histopathological scoring system (Heiple et al., 1987) after euthanasia at day 60.
Main Findings (relevant to PICO question)	Higher radiographic scores in the OG only group compared to the control group at day 30 and 60. Mean radiographic scores were similar (day 30 OG 3/10 vs control 1/10, day 60 OG 5/10 vs control 4/10), and there was a large amount of overlap between groups. Higher histopathology scores for the sum of histopathological criteria in OG only group compared to control group at day 60.
Limitations:	Very small sample size (n = 4 dogs for each group). There is no sample heterogeneity. No female dogs are included in the study. Unable to examine histologically throughout the study due to ethics constraints. A healthy dog population does not reflect hospitalised dogs. An osteotomy may not reflect natural fractures. Blinding for radiographic analysis not reported. Multiple comparisons in statistical analysis not accounted for. No power analysis. All authors (except one) worked in the university that conducted the study, and received financial support by the university. There is potential for bias. The study does not mention who conducted the statistical analysis or who estimated the radiographs. If the authors were involved, there is potential for bias.

**Baltzer et al. (2015):** 

Population:	Both male and female small dogs (≤ 6 kg, ~2.5 kg) from the Lois Bates Acheson Veterinary Teaching Hospital at Oregon State University, with a radial and / or ulnar fracture with open reduction and fixation with a bone plate, and radiographic evidence of complete fracture union. All dogs were free of other orthopaedic or neural abnormalities.
Sample size:	25 dogs, with 29 fractures (four dogs had two fractures each, either sequentially or simultaneously).
Intervention details:	Eight dogs were removed from the study due to having no radiographical evidence of healing, and 19/25 took part in the study. Open reduction and bone plate fixation with screws, with (n = 11 dogs, 13 fractures) or without (n = 8 dogs, 8 fractures) an omental graft (OG). Median time from fracture to surgical stabilisation was 14 days (range: 1–56 days) for all dogs. All OG dogs (n = 11 dogs, 13 fractures) underwent aseptic surgery to remove a 20 x 30 mm piece of the greater omentum. This was then placed over the bone plate and wrapped around the radius (and ulna when appropriate) on the surface of the bones. Splinted caudally for a minimum of 2–4 weeks.
Study design:	Retrospective clinical study.
Outcome studied:	Observation: Major complications (resulting in re-operation due to re-fracture, implant removal, osteopenia, wound dehiscence, screw protrusion through the skin, amputation). Minor complications (required veterinary attention but did not require surgery, for example pressure sores, swelling and oedema, discharge from the incision, persistent lameness, cold intolerance, licking of skin). Radiographic: Postoperative radiographs (obtained every 2–8 weeks until radiographic healing had occurred, defined by iso-opaque bridging bone callus) reviewed by investigators, assessed for apposition (defined by percentage overlap of fracture ends), alignment (categorised as good (< 5° of angulation or rotation abnormalities between radiohumeral joint and antebrachio-carpal joint) or poor [> 5°]), angulation, implant failure and osteopenia. Owner survey: Owners contacted by phone 3–52 months postoperatively and interviewed about the fracture, using a question set modified from the questionnaire by Worth et al. (2004).
Main Findings (relevant to PICO question)	Median time to complete radiographic healing was faster in OG dogs (70 days) compared to non-OG dogs (106 days). There was a higher occurrence of major complications in healing of the non-OG group (6/8 fractures) compared to the OG group (2/13 fractures), which resolved without treatment). There were minor complications in 8/13 OG fractures, and closure of the fracture site was more difficult than in the non-OG group. Only owners of dogs in the non-OG group reported that lameness had occurred.
Limitations:	Inconsistent time of follow-up radiographs meant no conclusion regarding rate of bone healing could be made. Small sample size. Retrospective nature of the study limited conclusions that could be made. Not all controlled variables were kept the same. For example, different techniques and surgeons were used, and 4/8 control (non-OG) dogs had a cancellous or corticocancellous bone graft. Incomplete data set due to loss of a dog (one from the OG group) to follow-up. Small size of dogs, which may not reflect the medium sized dog in this clinical scenario. A larger dog would result in more weight put on a radial fracture, which may impede healing. There is no sample heterogeneity. There is no statistical analysis. Radiographs were not evaluated by a published scoring system. No histological evaluation was performed due to study limitations. All authors (except one) worked in the university that conducted the study, and received financial support by the university. There is potential for bias.

## Appraisal, application and reflection

The primary aim of fracture repair is to restore the limb's normal function as efficiently and effectively as possible (Dvořák et al., 2000). While many fractures heal without complications and without the need of further corrective measures other than a splint, more severe fractures (such as comminuted or displaced, or those due to neoplasia) can lead to complications, such as delayed, mal- or non-union (Jackson & Pacchiana, 2004). There are various techniques used to repair fractures, such as external coaptation (very effective and economical, however very dependent on the type of case), implants (pins, nails, screws, plates), external fixators and adjuvants (such as autologous cancellous bone grafts) (Kumar et al., 2020; and Pozzi et al., 2021). These techniques are used to varying degrees, depending on the case and the practitioner's knowledge and capability. However, despite this range of techniques, complications still occur (Jackson & Pacchiana, 2004).

One novel approach, utilising free autologous greater omental grafts (OG) over the fracture site, has shown promise (Moran & Panje, 1987; and Thanoon, 2006). Among the many functions of the greater omentum, the presence of multipotent-mesenchymal stem cells allows it to differentiate into various tissues, such as bone, fat and cartilage (Di Nicola, 2019). Thanoon (2006), Saifzadeh et al., (2007) and others since, have found that by grafting part of the greater omentum onto bone fracture sites, they could help facilitate healing, as it increases vascularity and provides precursor cells for osteoblasts. Although not currently widely used in veterinary medicine, possibly due to its novel nature and abdominal surgery requirement, it could potentially prove to be greatly beneficial to aid healing of more complicated and severe bone fractures, where dire complications are more likely to occur. It has been reported to be useful in a variety of human surgeries, including vascular, neurological and orthopaedic surgery, and has been shown to reduce the occurrence of complications when healing more complex defects (Alagumuthu et al., 2006). As such, despite the novelty of the use of OG in veterinary medicine, its effectiveness demonstrated in human medicine indicates it requires further investigation.

Two of the studies (conducted by the same research group) (Bigham-Sadegh et al., 2012; and Karimi et al., 2013) were experimental case control studies, which involved selecting a group of healthy medium sized dogs and performing a radial midshaft osteotomy, followed by an omentectomy and subsequent grafting of omentum onto the fracture site for the experimental group. Both of these studies reported statistically significant findings, where the use of OG improved healing measured radiographically and histopathologically. Both papers reported higher radiographic scores in OG groups in the latter weeks, particularly from week 6 onwards. Bigham-Sadegh et al. (2012) found higher histopathological scores for union and cortical bone in OG group, and while Karimi et al. (2013) combined these scores, their OG group also scored higher.

However, there were a number of limitations to these. Both studies (Bigham-Sadegh et al., 2012; and Karimi et al., 2013) had very small sample sizes (n = 16 dogs), with only four dogs in each experimental and control group. Neither study was randomised. Blinding was reported only regarding the histopathological analysis, not for the radiographical analysis. It is worthwhile noting that the significance of the results was stronger for the radiographical analysis (not blinded) while it was weaker for the histopathological analysis (blinded), indicating the lack of blinding may have biased the results for both studies. They both used Kruskal-Wallis non-parametric ANOVA to assess statistical significance, as they had three experimental groups, and one control group. However, they did not account for multiple comparisons.

Dogs were euthanised and the bones collected for histopathological analysis at week 8 (Bigham-Sadegh et al., 2012) and at day 60 (Karimi et al., 2013). While there is evidence healing was both faster and greater in the OG groups compared to the control groups, extending the study until complete healing had occurred would have strengthened the level of evidence overall. Other limitations of these studies include the fact that a healthy dog population does not necessarily reflect hospitalised dogs. Hospitalised dogs may have concurrent illnesses that can compound and slow the healing process, and may have higher stress levels (and therefore a more severe stress leukogram), which can also interfere with the healing process. Adittionally, an osteotomy reflect a natural fracture. Natural fractures may be more complicated, are often caused by trauma (which would damage surrounding tissue), may not be well aligned, and may be non-sterile. The time between the fracture and surgical intervention in a natural fracture is usually greater than in an osteotomy as well.  In addition, there is no power analysis, there is no animal heterogeneity, it is not clear who conducted the statistical tests and the radiographic evaluation, and there is potential for bias due to the study funding. Further studies comparing the use of OG to no OG in a clinical setting would be beneficial. Consequentially, both these studies provide a weak level of evidence.

The final study was a retrospective clinical study (Balzer et al., 2015). Among other outcomes, they determined that the mean time to healing with OG was approximately two thirds of the time it took without OG (70 days versus 106 days). This was the only study to look at dogs with pre-occurring fractures to the radius in a clinical setting – the other studies were experimental with the fracture induced. They also reported on the differences in major and minor complications between the groups. They found that while the OG group had a higher occurrence of minor complications, the non-OG group had a far higher occurrence of major complications. Only 2/13 OG fractures had a more significant complication, however this resolved without treatment within 6 months. Most of the dogs in the non-OG group (6/8) however suffered major complications, all of which required surgery to fix. Note that dogs with screws in the ulna had worse complications. Ultimately, they concluded that OG reduced occurrence of major complications, however they noted that it was more difficult to close the incision over the fracture site with OG.

While the Baltzer et al. (2015) study is more relevant to the clinical scenario, there were many limitations to this study, particularly regarding its ability to answer the PICO question. They had a relatively small sample size, and furthering this, the dogs in each group had many different variables. For instance, some dogs broke their ulna instead of radius, or they broke both. The control group included some dogs that had other interventions (different types of grafts) as well as stabilisation. The time between fracture and stabilisation also varied significantly. All of these will undoubtably have impact on healing time and efficacy, however due to the presence of these variables, and the retrospective nature of the study, it is difficult to determine the extent of this.

Other limitations include the lack of statistical analysis, the incomplete data set due to loss of a dog from the OG group in follow-up and the lack of heterogeneity. Also, radiographs were not evaluated by a published scoring system; all authors (except one) worked in the university that conducted the study and received financial support by the same university, so there is potential for bias. No histopathological evaluation was performed, as these were client owned dogs undergoing treatment of their fractures where histopathological analysis was not necessary, and a retrospective study has no control over this. All of these led to the study also providing a weak level of evidence.

Aside from the limitations in each individual paper resulting in weakening of the evidence, there are also some issues when looking at the papers together. Perhaps the most significant is the difference in the type of studies – it is difficult to properly compare a retrospective study with experimental case control studies. The retrospective study (Baltzer et al., 2015) also used different surgical repair techniques, as well as different stabilisation methods. These may have affected healing, so it is best to look at this study separately to the experimental studies. Additionally, the two experimental studies are from the same research group. Due to this, there is potential for bias (specifically, observer bias) in their later study (Karimi et al., 2013) as they have some knowledge of results from a similar study. Overall, due to the limited number of studies, as well as the limitations to each study, there is weak evidence to address the PICO question and to recommend the use of OG in clinical practice.

When considering using OG in clinical practice, the possible benefits must be weighed against the risks involved. Using OG requires entering into the abdomen, which is an additional wound, more tissue handling, likely increased surgery time and increased risk of infection (Berzon, 1979). For routine fracture stabilisation, the use of OG is likely unnecessary, as it can possibly increase the risk for surgical complications. However, for the more complicated defects, the benefits are more likely to outweigh the risks of using OG. Batlzer et al. (2015) looked at complications and actually found fewer major complications using OG, however more minor complications. As such, it is ultimately up to the surgeon’s discretion as to whether OG is utilised in fracture repair.

Using OG to assist healing fractures in dogs has potential, however currently the evidence supporting its efficacy is weak. Many papers were found in the initial search, however very few addressed the PICO question. Further studies with blinding, randomisation, and a longer duration are needed. As well as this, studies that first do a power-analysis to determine the appropriate sample size needed to address the variability in the biological processes, as well as correction for variability with statistical methods, would be highly beneficial, and produce more reliable results. If more papers are written with the above recommendations, this will assist in strengthening the level of evidence to be able to recommend omental graft use in practice.

## Methodology

**Search strategy:** 

Databases searched and dates covered:	CABI: CAB Abstracts (Web of Science): 1910 – January 2023 Web of Science Core Collection: 1900 – January 2023 PubMed: 1900 – January 2023 Medline (via Ovid): 1946 – January 2023 Google Scholar: up to January 2023
Search strategy:	CAB Abstracts and Web of Science: Topic: ((canine OR canines OR canid OR canis OR dog OR dogs) AND (omentum OR greater omentum OR omental) AND (bone OR osseous OR ossein OR radii OR radius OR radiuses) AND (fracture OR osteotomy OR nonunion OR break OR broken) AND (graft OR transplant OR implant)) OR ((canine OR canines OR canid OR canis OR dog OR dogs) AND (omentum OR greater omentum OR omental) AND (bone OR osseous OR ossein OR radii OR radius OR radiuses) AND (graft OR transplant OR implant)) OR ((canine OR canines OR canid OR canis OR dog OR dogs) AND (omentum OR greater omentum OR omental) AND (fracture OR osteotomy OR nonunion OR break OR broken) AND (graft OR transplant OR implant)) OR ((canine OR canines OR canid OR canis OR dog OR dogs) AND (omentum OR greater omentum OR omental) AND (bone OR osseous OR ossein OR radii OR radius OR radiuses)) OR ((canine OR canines OR canid OR canis OR dog OR dogs) AND (omentum OR greater omentum OR omental) AND (fracture OR osteotomy OR nonunion OR break OR broken)) PubMed and Medline: ((canine OR canines OR canid OR canis OR dog OR dogs) AND (omentum OR greater omentum OR omental) AND (bone OR osseous OR ossein OR radii OR radius OR radiuses) AND (fracture OR osteotomy OR nonunion OR break OR broken) AND (graft OR transplant OR implant)) OR ((canine OR canines OR canid OR canis OR dog OR dogs) AND (omentum OR greater omentum OR omental) AND (bone OR osseous OR ossein OR radii OR radius OR radiuses) AND (graft OR transplant OR implant)) OR ((canine OR canines OR canid OR canis OR dog OR dogs) AND (omentum OR greater omentum OR omental) AND (fracture OR osteotomy OR nonunion OR break OR broken) AND (graft OR transplant OR implant)) OR ((canine OR canines OR canid OR canis OR dog OR dogs) AND (omentum OR greater omentum OR omental) AND (bone OR osseous OR ossein OR radii OR radius OR radiuses)) OR ((canine OR canines OR canid OR canis OR dog OR dogs) AND (omentum OR greater omentum OR omental) AND (fracture OR osteotomy OR nonunion OR break OR broken)) Google Scholar: “dog” “omentum” “bone” “fracture” “graft”
Dates searches performed:	03 Jan 2023

**Exclusion / inclusion criteria:** 

Exclusion:	Not primary research (such as reviews and CATs, case studies, conference abstracts, press articles). Not English. Duplicates. Does not answer the PICO question.
Inclusion:	English. Any clinical trial, experimental study or case series. Address the PICO question: Dogs with a radial fracture. Surgical intervention. Control group. Looking at reducing healing time.

**Search outcome:** 

Database	Number of results	Excluded – Did not answer the PICO question	Excluded – Not English	Excluded – Not primary research	Excluded – Duplicates	Total relevant papers
CAB Abstracts	40	38	0	0	1	1
Web of Science	43	41	0	0	2	0
PubMed	35	32	0	1	0	2
Medline	29	25	0	1	3	0
Google Scholar	687	677	3	4	3	0
Total relevant papers when duplicates removed						3

## ORCiD

Rhyanna Dietrich: https://orcid.org/0000-0003-0948-7161

## Conflict of interest

The author declares no conflicts of interest.

## Disclaimer

Knowledge Summaries are a peer-reviewed article type which aims to answer a clinical question based on the best available current evidence. It does not override the responsibility of the practitioner. Informed decisions should be made by considering such factors as individual clinical expertise and judgement along with patient’s circumstances and owners’ values. Knowledge Summaries are a resource to help inform and any opinions expressed within the Knowledge Summaries are the author’s own and do not necessarily reflect the view of the RCVS Knowledge. Authors are responsible for the accuracy of the content. While the Editor and Publisher believe that all content herein are in accord with current recommendations and practice at the time of publication, they accept no legal responsibility for any errors or omissions, and make no warranty, express or implied, with respect to material contained within. For further information please refer to our Terms of Use.
